# The Evolution of Data Fusion Methodologies Developed to Reconstruct Coronary Artery Geometry From Intravascular Imaging and Coronary Angiography Data: A Comprehensive Review

**DOI:** 10.3389/fcvm.2020.00033

**Published:** 2020-03-31

**Authors:** Yakup Kilic, Hannah Safi, Retesh Bajaj, Patrick W. Serruys, Pieter Kitslaar, Anantharaman Ramasamy, Vincenzo Tufaro, Yoshinobu Onuma, Anthony Mathur, Ryo Torii, Andreas Baumbach, Christos V. Bourantas

**Affiliations:** ^1^Department of Cardiology, Barts Heart Centre, Barts Health NHS Trust, London, United Kingdom; ^2^Institute of Cardiovascular Sciences, University College London, London, United Kingdom; ^3^Centre for Cardiovascular Medicine and Device Innovation, Queen Mary University London, London, United Kingdom; ^4^Faculty of Medicine, National Heart & Lung Institute, Imperial College London, London, United Kingdom; ^5^Department of Radiology, Leiden University Medical Center, Leiden, Netherlands; ^6^Erasmus University Medical Center, Rotterdam, Netherlands; ^7^Department of Mechanical Engineering, University College London, London, United Kingdom

**Keywords:** hybrid intravascular imaging, data fusion methodologies, 3D reconstruction, coronary artery modeling, coronary angiography

## Abstract

Understanding the mechanisms that regulate atherosclerotic plaque formation and evolution is a crucial step for developing treatment strategies that will prevent plaque progression and reduce cardiovascular events. Advances in signal processing and the miniaturization of medical devices have enabled the design of multimodality intravascular imaging catheters that allow complete and detailed assessment of plaque morphology and biology. However, a significant limitation of these novel imaging catheters is that they provide two-dimensional (2D) visualization of the lumen and vessel wall and thus they cannot portray vessel geometry and 3D lesion architecture. To address this limitation computer-based methodologies and user-friendly software have been developed. These are able to off-line process and fuse intravascular imaging data with X-ray or computed tomography coronary angiography (CTCA) to reconstruct coronary artery anatomy. The aim of this review article is to summarize the evolution in the field of coronary artery modeling; we thus present the first methodologies that were developed to model vessel geometry, highlight the modifications introduced in revised methods to overcome the limitations of the first approaches and discuss the challenges that need to be addressed, so these techniques can have broad application in clinical practice and research.

## Introduction

Invasive coronary angiography is the reference standard for assessing the extent and severity of coronary artery disease (CAD) which is a leading cause of death in the developed and developing world (1). This modality however provides only a two dimensional (2D) representation of lumen anatomy and thus it has limitations in quantifying luminal stenosis especially in the cases of foreshortening and vessel overlapping (2). Moreover, coronary angiography cannot assess 3D vessel geometry and accurately quantify lesion length. To address these limitations computerized based methodologies have been developed that allow 3D reconstruction of the coronary artery anatomy from two or multiple angiographic views (2–4); These approaches enable assessment of vessel geometry and accurate estimation of lesion length and stenosis severity (2, 3). But on the other hand they do not allow visualization of plaque characteristics which determine plaque evolution and vulnerability (5–7).

**Figure 1:**
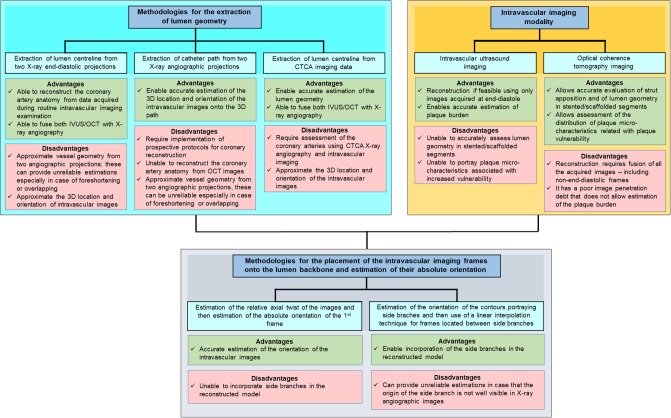
Advantages and limitations of different intravascular imaging modalities and algorithms proposed in different reconstruction methodologies for the modelling of coronary artery geometry.

Over the last few decades intravascular imaging catheters have been designed which can be advanced into the coronary arteries to obtain high-resolution cross-sectional images of the vessels. This enables comprehensive visualization of the lumen and plaque pathology. Intravascular ultrasound (IVUS) and optical coherence tomography (OCT) were the first invasive imaging techniques that were used to study plaque pathobiology and provided unique insights about the mechanisms that regulate plaque evolution. Validation studies using histology as the gold standard have demonstrated the advantages but also the limitations of IVUS and OCT in assessing plaque characteristics and led research toward the development of novel invasive imaging modalities that were able to overcome the drawbacks of the first techniques (8–11). Near infrared spectroscopy (NIRS), photoacoustic imaging (IVPA), near infrared fluorescence imaging (NIRF), and time resolved fluorescence spectroscopy (TRFS) imaging are some of these invasive imaging techniques that were introduced to provide additional information about vessel pathology and biology. These modalities have been combined with IVUS or OCT in hybrid intravascular catheters that are currently undergoing clinical or preclinical evaluation and are expected to provide unique mechanistic insights about atherosclerotic evolution (4, 11). A limitation of these hybrid techniques is that they are unable to portray the 3D vessel geometry. To overcome this drawback several data fusion methodologies have been developed that can retrospectively process intravascular imaging and coronary angiography to generate 3D realistic models of vessel architecture (Table 1). These models allow comprehensive visualization of the distribution of the plaque and can be processed with computational fluid dynamic (CFD) techniques to estimate plaque structural stress (PSS) and endothelial shear stress (ESS), which determine atherosclerotic evolution and predict future events (12, 13). The aim of this review article is to provide a comprehensive overview of the data fusion methodologies developed for vessel modeling; we describe the approaches introduced to fuse intravascular imaging and angiographic data to model vessel geometry, present the modifications that were made to optimize vessel reconstruction and facilitate their application in research, and discuss the challenges that should be overcome so that these approaches can be broadly used in the study of atherosclerosis (Figure 1).

**Table 1:** The evolution of 3D reconstruction methodologies.

**Methodology**	**Accurate extraction of lumen geometry**	**Accurate estimation of the orientation of the intravascular imaging frames**	**Extensive validation**	**Side branch reconstruction**	**Capable to fuse non-gated intravascular images**	**No need for prospective imaging protocol**	**Reliable reconstruction of stent architecture**
Klein et al. (22)	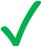	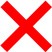	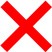	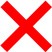	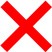	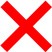	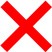
Lengyel et al. (23)	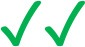	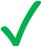	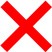	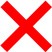	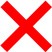	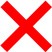	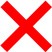
Wahle et al. (27)	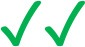	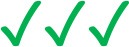	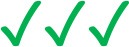	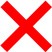	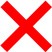	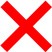	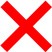
Slager et al. (32)	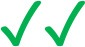	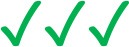	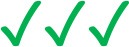	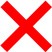	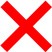	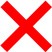	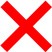
Bourantas et al. (29)	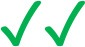	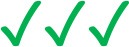	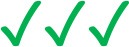	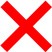	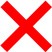	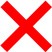	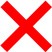
Giannoglou et al. (28)	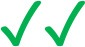	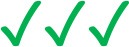	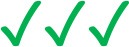	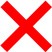	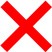	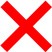	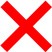
Tu et al. (83)	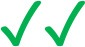	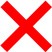	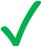	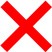	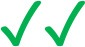	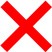	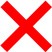
Van der Giessen et al. (58)	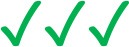	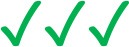	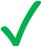	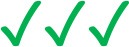	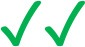	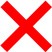	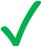
Bourantas et al. (43)	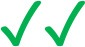	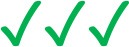	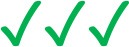	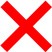	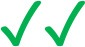	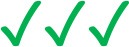	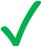
Li et al. (57)	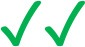	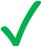	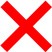	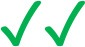	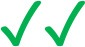	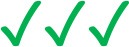	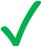
Li et al. (77)	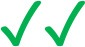	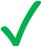	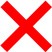	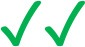	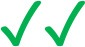	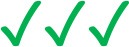	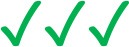

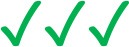
, excellent ability of the modality to detect the specific feature; 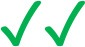, moderate ability of the modality to detect the specific feature; 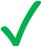, weak ability of the modality to detect the specific feature; 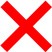, the modality is unable to detect the specific feature.

*Advantages and limitations of the data fusion imaging techniques developed to reconstruct coronary artery anatomy*.

## First Attempts for Coronary Artery Reconstruction

Roelandt et al. were the first that attempted to reconstruct coronary artery anatomy from IVUS imaging data (14). They assumed that the catheter pull-back trajectory was a straight line and then stacked the IVUS frames perpendicularly onto the catheter trajectory to create a 3D model of the vessel wall that had a cylindrical shape. The final model enabled evaluation of the plaque volume and facilitated the development of methodologies that would allow segmentation of the lumen and outer vessel wall borders (15). A limitation of this approach is that the IVUS model included frames acquired during the entire cardiac cycle; however the vessel is moving during the cardiac cycle and as a consequence the relative lateral and longitudinal position of the IVUS probe with regards to the lumen changes (16). These changes result in the so called “saw tooth” artifact in the 3D model that is more prominent in normal vessels with increased diameter and preserved compliance. To address this pitfall electrocardiographic (ECG)-gated pull-back systems and methodologies for a retrospective gating of the ECG data were proposed; these approaches enabled selection of frames acquired at a specific period of the cardiac cycle (i.e., at the end-diastole) which were then used to reconstruct the vessel geometry (17, 18).

A similar approach was also introduced to reconstruct coronary artery anatomy from OCT data (19). The obtained models allowed evaluation of the distribution of the plaque, of stent apposition, accurate detection of stent fracture and visualization of the orifice of the side branches (20). Today commercially available software have been developed for this purpose and have been incorporated in the OCT systems enabling real time representation of vessel morphology, evaluation of the procedural results and optimal treatment planning.

Despite the undoubted role of these reconstruction methodologies in the clinical arena, they have significant limitations as they are unable to portray coronary artery geometry, evaluate the distribution of the plaque onto the vessel and accurately quantify plaque volume especially in the case of increased curvature where neglecting vessel curvature can lead to an underestimation of the plaque volume by 5% (21).

## Fusion of Coronary Angiography and Intravascular Ultrasound

In 1992 Klein et al. for the first time suggested fusion of IVUS and X-ray angiography for a more reliable assessment of vessel architecture (22). The proposed methodology included the segmentation of the IVUS images, the extraction of the luminal centerline from two angiographic projections and the placement of the detected contours onto the luminal centerline. A limitation of the proposed methodology is that it was unable to correctly orientate the IVUS borders onto the luminal centerline. Lengyel et al. (23) in 1995 overcame this limitation by using anatomical landmarks (i.e., side branches) that were visible in both IVUS and angiographic images to estimate the rotational orientation of the IVUS frames. This methodology was easy to use and appeared able to provide geometrically correct reconstruction; however it did not have applications in the clinical arena because it was not validated in detail (23).

A year later Shekhar et al. proposed an alternative approach for coronary reconstruction; the authors used an ECG-gated pull-back device for IVUS pull-back and acquired numerous biplane angiographic images during the pull-back so as to identify in X-ray images the position of the IVUS-catheter tip at each end-diastolic frame (24). They then extracted the IVUS catheter path from the biplane angiographic images and placed each frame onto the path in the corresponding position; each frame was then rotated at an angle so as its projections onto the angiographic images to best match with the luminal silhouette in these projections. *In vivo* validation of the developed methodology using X-ray angiography as the gold standard demonstrated that it provides accurate coronary modeling; however the time consuming protocol and increased radiation required for coronary reconstruction did not allow this method to have applications in the clinical arena (25).

**Figure 2:**
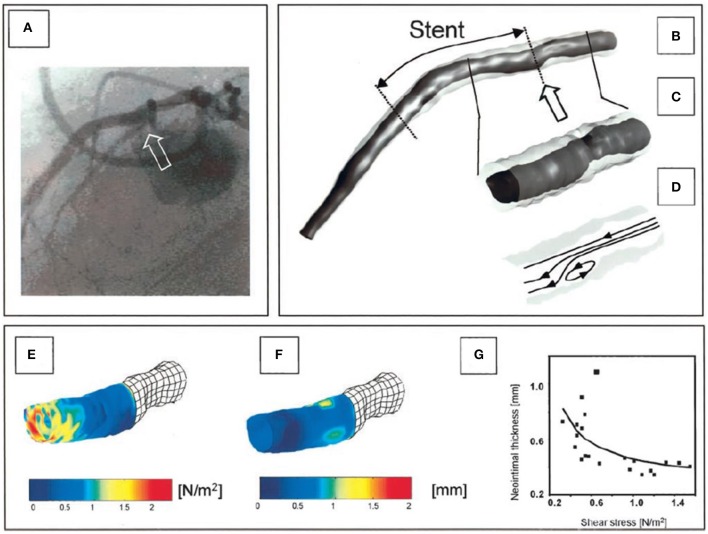
One of the first applications of *in vivo* 3D-reconstruction modeling that examined the role of ESS on neointima formation. **(A)** shows an angiographic projection of the left anterior descending coronary artery treated with bare metal stents; the arrow indicates a step-up at the proximal segment of the stent. **(B)** shows the coronary artery model post stent implantation reconstructed from the fusion of X-ray angiography and IVUS; the outer vessel wall surface is shown in a semi-transparent fashion. The arrow indicates the step-up location noted in the angiographic images; **(C)** shows a magnified view of that segment while **(D)** portrays the blood flow streamlines estimated after blood flow simulation; a recirculation zone is noted at the step-up site. At that location low ESS are noted **(E)** that co-localize with increased neointima proliferation noted in the vessel model at 4 months follow-up **(F)**. A significant inverse association was reported between ESS and neointima thickness at follow-up **(G)**. The figure was obtained with permission from Thury et al. (30).

**Figure 3:**
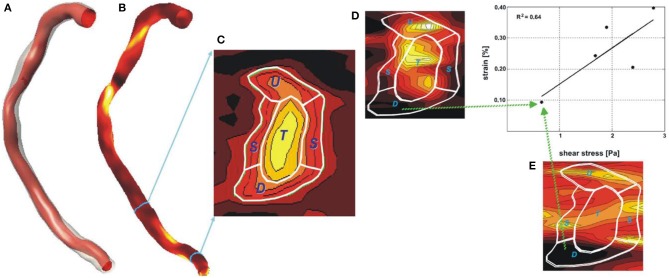
Reconstruction of a right coronary artery from IVUS and angiographic data with the lumen surface portrayed in a red color and the outer vessel wall in a semi-transparent fashion **(A)**. In **(B)** the plaque distribution is displayed in a color-coded map (the red-brown color corresponds to low plaque thickness and yellow to increased plaque). **(C)** shows the distribution of the plaque in a stenotic segment in 2D. The symbols U, L, D, and T denote the upstream the lateral, the downstream shoulder and the throat of the plaque, respectively. **(D)** illustrates in 2D the plaque strain distribution and **(E)** the ESS distribution. It is obvious that there is a good correlation between vessel wall strain and ESS implying an interaction between these two variables. Image obtained with permission and modified from Gijsen et al. (31).

Conversely the methodology of Laban et al. presented in 1995 required only two biplane angiographic projections for coronary artery reconstruction enabling its broad application in the study of atherosclerosis (26). The first biplane projection should portray a calibration object, the IVUS catheter and the lumen silhouette—obtained during diluted contrast agent injection—before the beginning of the IVUS pull-back while the second the calibration object the IVUS catheter and the lumen silhouette at the end of the pull-back. These projections were used to extract the IVUS catheter path where it was assumed that it corresponded to the IVUS trajectory during the pull-back. The end-diastolic frames acquired during IVUS imaging were identified and segmented and the detected contours were placed perpendicularly onto the catheter path. The Frenet-Serret formula was then used to estimate the relative axial twist of the IVUS frames. Their absolute orientation was estimated by comparing the projections of these frames and the projection of the reconstructed path onto the angiographic images with the lumen and path silhouette in these X-ray images. Similar approaches for coronary artery reconstruction were proposed by Wahle et al., Gianoglou et al. and Bourantas et al. who proposed a more robust methodology for the catheter path extraction from coronary angiography (27–29). The above approaches were extensively validated in phantom models, *in vivo* and *ex vivo* and were broadly used in the research arena to study plaque strain distribution and the implications of the local hemodynamic forces on plaque formation destabilization and rupture in native and stented segments (Figures 2, 3) (27–29, 31–35).

However, a limitation of the above methodologies is the fact they require the implementation of a specific protocol in the catheterization laboratory that includes X-ray imaging of calibration objects and acquisition of biplane angiographic images that would allow visualization of the IVUS catheter and the lumen silhouette at the beginning and the end of the pull-back. To enhance the application of coronary modeling in research, Bourantas et al. proposed an updated reconstruction methodology that includes the extraction of the luminal centerline of the studied vessel from two angiographic projections—as suggested by Leyngyel et al. (23); the identification and segmentation of the end-diastolic IVUS images, the placement of the IVUS borders perpendicularly onto the vessel centerline, the estimation of their relative twist using the sequential triangulation algorithm and then the use of anatomical landmarks seen in both IVUS and X-ray imaging to define the orientation of the 1st IVUS frame (36). Validation of this approach using the conventional “catheter-path” reconstruction methodology (29) in 22 patients (27 vessels) demonstrated that this new approach allows reliable representation of vessel geometry, quantification of the luminal dimensions and atheroma burden and accurate estimation of the ESS distribution (36). This approach enabled for the first time coronary artery reconstruction using data acquired during conventional IVUS imaging and today it has been extensively used to process imaging data obtained in large intravascular imaging studies of coronary atherosclerosis such as the PROSPECT and IBIS 4 studies and examine the implications of the local hemodynamic forces on plaque progression and destabilization in native vessels and on neointima healing and composition in drug eluting and bare metal stents (37–39).

The “centerline” methodology presented by Bourantas et al. may have broadened the application of computational modeling in the study of atherosclerosis but it also has a major limitation as it does not incorporate side branches in the final model which appear to alter flow patterns and affect ESS estimations (40). To overcome this problem Samady et al. proposed inclusion of side branch geometry in the final model (41). Side branch reconstruction in this methodology is performed by taking into account its orifice in IVUS images and the side branch geometry extracted from the angiographic images. This approach has been used to examine the effect of the ESS and oscillatory ESS on plaque composition and morphology but is yet to be validated in detail (41, 42).

## Fusion of Coronary Angiography and Optical Coherence Tomography Imaging

The fusion of IVUS and X-ray imaging may have provided unique opportunities for assessing vessel geometry, quantifying plaque burden and examining the effect of the local hemodynamic forces on plaque progression. However, it has failed to accurately assess the interplay between ESS and plaque micro-characteristics associated with increased vulnerability and the estimation of flow patterns following stent/scaffold implantation. To overcome these limitations and examine the implications of local hemodynamic forces on plaque destabilization and rupture, fusion of OCT and coronary angiography has been proposed. Bourantas et al. were the first that used OCT-based reconstruction to assess ESS distribution in a ruptured plaque (43). The culprit vessel was imaged using a M3 OCT system (LightLab Imaging Inc., Westford, MA, USA) that was pulled-back at a constant speed of 3 mm/s. From these images the end-diastolic frames were selected, using a view mixer that allowed simultaneous visualization of the ECG and OCT sequence, and these frames were then co-registered with the angiographic data using an established methodology that was proposed for the fusion of X-ray angiography and IVUS (29). Blood flow simulation was performed in the obtained model and the ESS was estimated. High ESS was noted in the region of the ruptured plaque; these findings are in line with the results reported in IVUS-based reconstructions and highlight the potential implications of flow patterns on plaque destabilization and rupture (44, 45).

The first methodology for geometrically correct reconstruction of the coronary artery anatomy from frequency domain (FD)-OCT and X-ray angiography was proposed by Athanasiou et al. (46) and validated by Papafaklis et al. (47). FD-OCT-based reconstruction poses a challenge, as in contrast to the IVUS-based modeling where only end-diastolic frames are used, in FD-OCT modeling all the frames have to be included. This is because the high pull-back speed (18–40 mm/s) in FD-OCT results in a very small number of end-diastolic images. Placement of non-end-diastolic frames onto the catheter path—as previous approaches suggested (27–29, 32)—would result in motion artifacts and a rugged luminal surface because of the relative lateral movement of the OCT catheter in the lumen during the cardiac cycle. In order to address this challenge Athanasiou et al. (46) proposed the use of the lumen centerline, extracted from two end-diastolic angiographic projections, for the placement of the OCT contours. Then the authors used the sequential triangulation algorithm and the origin of side branches to estimate the absolute orientation of the OCT frames. A similar approach for OCT-based reconstruction has been proposed by Toutouzas et al. (48). *In vivo* validation of the “centerline” methodology using IVUS-based reconstruction as the gold standard showed that OCT-based modeling is effective in assessing vessel geometry and estimating ESS distribution (47). Over the last years this methodology has been extensively used to examine the association between local hemodynamic forces and plaque micro-characteristics (49, 50), assess the implications of flow patterns on neointima and neo-atherosclerotic lesion formation and rupture (51, 52), and evaluate the effect of different stent/scaffold designs on the local hemodynamic environment (53–55).

The above OCT-based reconstruction methodology may have significant applications in the research arena but it also has two significant limitations: (1) it is not able to correct the geometrical error caused by the longitudinal movement of the OCT catheter within the vessel during the cardiac cycle (16, 56) and (2) the obtained 3D models do not incorporate vessel's side branches. To address these drawbacks Li et al. proposed a modified approach for coronary artery reconstruction (57). This method suggested the use of 3D QCA to reconstruct the segment that was assessed by OCT and its side branches and then fuse the OCT images with the 3D QCA model. The orifices of the side branches were identified in the OCT images and this information was used to identify the longitudinal position of the OCT frames onto the 3D QCA model and estimate their absolute orientation. For the frames located between the side branches an interpolation technique was used to estimate their location and absolute orientation. A CFD analysis of the reconstructed models showed that the incorporation of the side branches had a significant effect on the ESS distribution with the average ESS being 4.64 Pa lower in models that included the side branches comparing to those that did not contain the side branches (*P* < 0.0001). Although this approach appears superior to others, previously reported in the literature, it does have limitations as it makes two assumptions; more specifically: (1) it implements an interpolation technique to place on the lumen centreline the OCT frames between those portraying side branches; this assumption cannot correct the error caused by the longitudinal motion of the OCT catheter that is increased at the beginning of the systole, and (2) it uses the origin of the side branches, which cannot be always accurately assessed in two angiographic projections, to estimate the rotational orientation of the OCT contours.

Moreover, this approach has not been thoroughly validated and therefore it is unclear what is the effect of the above limitations on vessel reconstruction and ESS computation.

## Fusion of Computed Tomography Coronary Angiography and Intravascular Imaging

The methodologies developed to reconstruct the coronary artery anatomy from X-ray angiography and intravascular imaging data rely on the extraction of the lumen centerline or the catheter path from two angiographic projections. The angle difference between the two projections as well as the presence of vessel foreshortening in these projections can affect the efficacy of these approaches in assessing vessel geometry. Moreover, as it was stated above, the origin of the side branches is likely to not be well visible in the projections used for coronary artery reconstruction and this can affect the accurate estimation of the absolute orientation of the intravascular images on the 3D model. These limitations can be overcome by the use of CTCA which provide 3D images of the coronary artery tree.

In 2010 van der Giessen et al. were the first to propose the fusion of CTCA and IVUS imaging to reconstruct the coronary arteries (58). This approach includes the following steps: (1) extraction of the lumen centerline from CTCA, (2) creation of CTCA cross sectional images that are perpendicular to the side branches, (3) identification of matched frames between end-diastolic IVUS and CTCA images, (4) placement of the IVUS frames showing side branches perpendicularly onto the lumen centerline in the corresponding locations in CTCA, (5) estimation of their absolute orientation using these branches, and (6) placement of the end-diastolic IVUS frames located between the frames with identifiable landmarks onto the vessel centerline using linear interpolation and estimation of their rotational orientation using spherical interpolation (Figure 2). This approach has significant advantages as it allows: (1) full representation of the coronary artery anatomy and geometry including side branches, (2) accurate extraction of vessel architecture from the 3D CTCA imaging data, and (3) reliable orientation of the intravascular images.

This approach has been used to evaluate the role of ESS on vessel wall healing following bioresorbable scaffold implantation and investigate the role of multidirectional ESS on the development of advanced atherosclerotic plaques in pig models (59, 60). The accurate co-registration of intravascular imaging and CTCA also offers the potentiality for a direct comparison of these two techniques and thus detailed evaluation of the ability of non-invasive imaging in assessing the lumen and outer vessel wall dimensions and characterizing plaque morphology (58, 61–63). A major limitation of this approach is that coronary reconstruction requires CTCA, coronary angiography and intravascular imaging data. Moreover, similar to the other reconstruction approaches this methodology is laborious and time consuming as human interaction is needed in most of the reconstruction steps and it has not been validated yet.

## Software Developed for Coronary Artery Modeling

To enhance the research applicability of the 3D reconstruction methodologies mentioned above, user—friendly systems have been developed that operate in a user-friendly environment and expedite coronary reconstruction (Figure 3). The first software was developed by Wahle et al. and incorporated the algorithms of the methodology developed by Wahle et al. in 1999 (27). To visualize the final models the authors used the standardized Virtual Reality Modeling Language (VRML) and designed a module where the operator could assess the luminal and the media—adventitia surface and estimate the plaque burden distribution since the plaque thickness was color coded displayed (64). Moreover, this system allowed evaluation of vessel curvature that was also portrayed using a color coded map and incorporated a 3D graphical user interface that enabled virtual endoscopy of the reconstructed vessel. In addition, the software included a module for blood flow simulation and computation of the ESS. Despite these unique advantages this system had limited application in research as it required the implementation of prospective protocol for data acquisition and increased time for coronary artery reconstruction.

ANGIOCARE (Figure 5) was the 2nd system developed for the fusion of intravascular imaging and coronary angiography (Figures 3A–D) (65). This software incorporated the methodology for arterial reconstruction proposed by Bourantas et al. (29) and a module for the segmentation of the IVUS images (66). In addition, ANGIOCARE included a 3D photorealistic visualization platform that allowed comprehensive representation of the reconstructed model, visual inspection of vessel morphology and evaluation of the distribution of the plaque (depicted in a color—coded map). In addition, the developed module allowed the operator to interact with the model select a segment and obtain quantitative information (i.e., lesion length, plaque volume, minimum luminal area, the reference luminal area, etc.) that could be used for PCI planning. A limitation of ANGIOCARE is the fact that it did not allow blood flow simulation and estimation of the ESS distribution.

IVUSAngio tool is the only freely available software for the fusion of IVUS and angiographic imaging data (67). The software incorporated the methodology of Giannoglou et al. (67) and included modules designed for the segmentation of IVUS, the extraction of the catheter path and the fusion of the imaging data and included a visualization platform where the operator could interact with the reconstructed vessel, review and examine model architecture and plaque distribution, identify the location of each frame onto the model and inspect the 3D model from inside using a fly through camera.

University of Leiden and Medis Medical Imaging Systems BV have recently developed a novel software for coronary reconstruction (Figures 3E–H). The software requires as an input the 3D QCA model—including vessel's side branches—of the segment assessed by IVUS/OCT that was reconstructed by the QAngio XA 3D software (Medis Medical Imaging Systems, Leiden, The Netherlands) and the IVUS/OCT borders segmented using the QCU-CMS software (Division of Image Processing, Department of Radiology, Leiden, The Netherlands, Leiden, The Netherlands). It has incorporated the algorithms of the methodology proposed by Li et al. (57) in a user-friendly environment and allows seamless reconstruction of vessel architecture and generation of 3D models that are stored in an .stl or .iges format. The latter software is currently used to process data collected in large scale prospective intravascular imaging studies of coronary atherosclerosis and assess the role of ESS distribution in predicting atherosclerotic disease progression and lesions with a vulnerable phenotype that will cause events.

## Discussion

Fusion of intravascular imaging data and X-ray angiography or CTCA imaging has enabled accurate reconstruction of coronary artery geometry and the generation of 3D models that can be processed with CFD techniques to evaluate vessel flow patterns and examine the effect of the hemodynamic forces on plaque evolution. These models have been enriched our understanding about the mechanisms that regulate atherosclerotic disease progression enabling more accurate prediction of lesions that are likely to progress and cause events (35, 37, 38).

The PREDICTION study which was the first prospective clinical study that used at scale fusion of intravascular imaging and X-ray angiography to assess the ESS distribution and highlighted the prognostic value of ESS in predicting plaque progression, thereby creating hope that an invasive assessment of plaque morphology combined with CFD analysis could enable accurate detection of vulnerable plaques (35). The findings of this study led research toward the development of easy to use methodologies that will be able to generate accurate representation of vessel geometry by fusing IVUS or OCT data with X-ray imaging data acquired during a conventional coronary angiography. These approaches have been used to retrospectively analyze intravascular imaging data acquired in large scale imaging studies of coronary atherosclerosis. A CFD analysis of the data acquired in the PROSPECT study showed that lesions with a high-risk morphology that were exposed to low ESS were likely to progress and cause cardiovascular events. This study also showed that the patients who had lesions with an unfavorable plaque morphology and physiology were the most vulnerable and at a high-risk to suffer a cardiovascular event (37). Retrospective fusion of IVUS, OCT and coronary angiography has also been used to process the data acquired in the IBIS 4 study—a multicenter study that utilizes serial virtual histology(VH)-IVUS and OCT imaging to assess the implications of aggressive treatment with Rosuvastatin on plaque phenotype—and examine the predictive accuracy of ESS patterns and plaque characteristics assessed by multimodality imaging in detecting segments that were likely to exhibit disease progression at 13 months follow-up (38, 68). In this study low ESS and VH-IVUS-derived but not OCT-derived plaque characteristics were predictors of disease progression. The findings of this analysis casted doubts about the efficacy of multimodality imaging in detecting vulnerable plaques. A limitation of this study was the small number of the studied vessels and the absence of events that did not allow us to examine the potential of multimodality imaging combined with CFD-modeling in detecting plaques that will cause events. Future studies are expected to use hybrid intravascular imaging to thoroughly assess plaque morphology and pathology and fuse these data with X-ray angiography or CTCA to determine ESS distribution in order to accurately predict vulnerable plaques. Studies combining NIRS-IVUS and X-ray or CTCA imaging to examine the association of ESS and plaque composition and evaluate its role on plaque progression have been recently reported (60, 69); data acquired in the future by the combined IVUS-OCT imaging catheter or by the hybrid NIRS-OCT or the IVPA-IVUS catheter are anticipated to be merged with X-ray or CTCA images to assess more accurately the interplay between plaque phenotype and plaque physiology. Moreover, models obtained by NIRF-IVUS or NIRF-OCT imaging combined with X-ray or CTCA imaging data, are anticipated to allow assessment of the role of ESS distribution on vascular biology and plaque inflammation.

The developed user-friendly software that included established data fusion algorithms are expected to facilitate research in the field and allow more complex simulations and complete assessment of vessel physiology. Cumulative data have highlighted the role of PSS on plaque destabilization and its value in predicting vulnerable lesions (70–72). The studies however that examined the prognostic implications of PSS focused on the analysis of IVUS cross-sectional images and did not take into account 3D vessel geometry. Further advances in software design and incorporation of plaque composition in the 3D models-derived by hybrid intravascular imaging techniques or polarized OCT (73) are expected to enable accurate assessment of PSS and evaluation of the synergetic effect of ESS and PSS on vulnerable plaque formation, destabilization and rupture (74).

**Figure 4:**
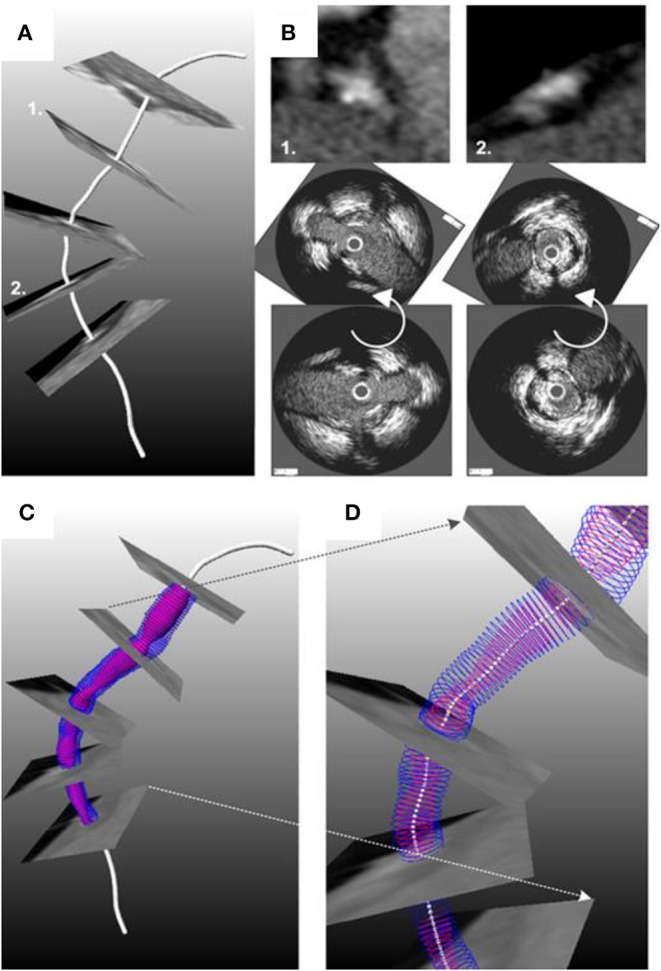
Methodology developed for the reconstruction of the coronary artery anatomy from CTCA and intravascular imaging data. **(A)** The luminal centerline is extracted from the CTCA imaging data and then CTCA cross-sectional images are generated perpendicularly to the centerline. **(B)** The CTCA images are matched with the IVUS images using anatomical landmarks that are seen in both IVUS and CTCA; the IVUS images are placed onto the luminal centerline and then the landmarks are used to estimate their absolute orientation. An interpolation technique is used to estimate the location and orientation of the frames located between side branches. The final model is shown in **(C,D)**. The figure was obtained with permission from Gijsen et al. (78).

Recent reports have highlighted the need to refine the methodologies for the reconstruction of vessel architecture especially in stented segments (75–77). Advanced methodologies have been presented lately (Figures 4, 6) that are able to separately reconstruct stent geometry and lumen surface and then fuse these models to generate a final lumen-stent object. These approaches are anticipated to provide more realistic representation of lumen architecture in stented segments and enable reliable evaluation of ESS distribution especially in the case of strut malapposition and at the orifice of the side branches where the protruded struts cause flow disturbances. These methods will be used in the future to conduct complex CFD analyses that will take into account the non-Newtonian behavior of the blood to precisely compute shear rate and stress distribution, quantify flow disturbances and identify areas that are exposed to an unfavorable hemodynamic milieu and are at risk of restenosis and stent/scaffold thrombosis (79–81). Moreover, these reconstruction approaches are expected to have value in the evaluation of the hemodynamic implications following implantation of different stent/scaffold designs and be extensively used to optimize stent configuration and develop revisions that will create a favorable hemodynamic environment following their implantation (54, 55, 82).

**Figure 5:**
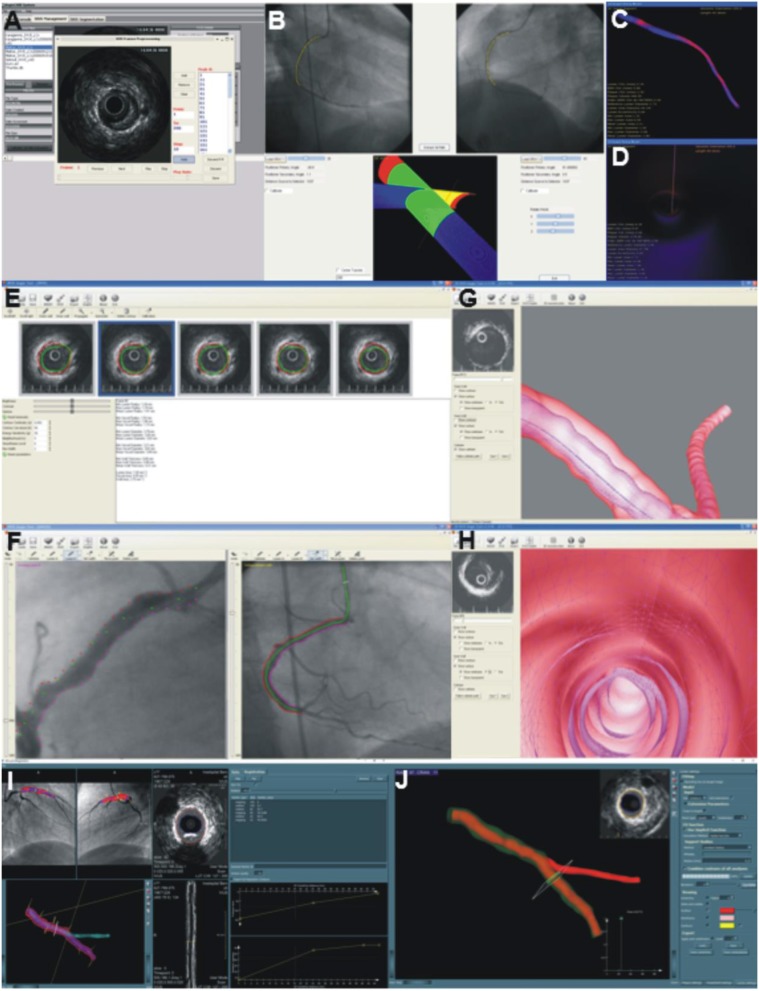
Software developed to reconstruct the coronary artery anatomy in a user-friendly environment. **(A–D)** portray a snapshot of the ANGIOCARE software. **(A)** illustrates the module developed for the pre-processing and analysis of intravascular imaging data, while **(B)** the module designed for the extraction of the catheter path from two angiographic projections. Finally, **(C,D)** show the platform designed for the visualization of the 3D model. The operator can appreciate the lumen geometry **(C)**, assess plaque distribution portrayed in a color-coded map (blue indicates no plaque and red indicates increased plaque burden) and assess the lumen morphology from inside **(D)**. **(E–H)** portray snapshots of the IVUSAngio tool. **(E)** shows the module for IVUS analysis, **(F)** the tool designed for the catheter path extraction, **(G)** illustrates the 3D model with the media-adventitia shown in a semi-transparent fashion, enabling evaluation of the distribution of the plaque burden and **(H)** portrays an endoscopic view of the lumen morphology. Finally, **(I,J)** illustrate snapshots of the software designed by Leiden University for the reconstruction of coronary artery anatomy. The annotated intravascular imaging data and the 3D-QCA model are imported and fused. The operator can identify in the lumen centerline the location of frames portraying side branches and use these to estimate their rotational orientation **(I)**. The reconstructed lumen is then fused with the side branch model obtained by 3D-QCA to generate the final vessel geometry. **(J)** shows the reconstructed vessel; the outer vessel wall is shown in a semi-transparent fashion, which allows evaluation of the distribution of the plaque.

**Figure 6:**
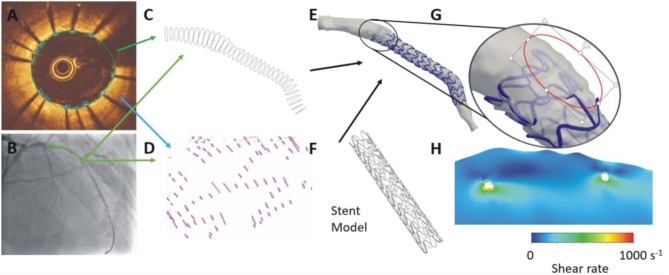
Advanced methodology proposed for the reconstruction of stented segments. In each OCT frame the lumen border is detected and the location of the stent struts is annotated **(A)**. The OCT contours are placed perpendicularly onto the lumen centerline **(B)** extracted by two angiographic projections and the location of the side branches are used to estimate their absolute orientation **(C)**. A similar approach is used to estimate the location of the struts in 3D space **(D)**. A methodology that relies on the location of the stent struts in 3D space and on a priori knowledge of stent architecture is used to reconstruct the stent geometry that is fused with the lumen geometry to reconstruct the stented segment **(E,F)**. This approach enables accurate reconstruction of the protruded or malapposed struts **(G)** and evaluation of their implications on the local hemodynamic forces **(H)**.

## Conclusion

Fusion of intravascular imaging and angiographic or CTCA imaging data allows generation of 3D models that accurately portray the vessel geometry and enable evaluation of plaque composition. These approaches have been extensively used to examine the implications of flow patterns on atherosclerotic disease progression and stent/scaffold thrombosis. Further advances in intravascular imaging, catheter design and the development of methodologies that will allow estimation of the distribution of different plaque components on the 3D plaque, and accurate reconstruction of stent architecture are expected to provide a complete and detailed evaluation of luminal geometry and coronary artery pathology. They are also expected to permit more precise quantification of the local hemodynamic forces, better prediction of plaque evolution, and optimization of focal therapies developed for the treatment of culprit or vulnerable lesions.

## Author Contributions

All authors listed have made a substantial, direct and intellectual contribution to the work, and approved it for publication.

### Conflict of Interest

The authors declare that the research was conducted in the absence of any commercial or financial relationships that could be construed as a potential conflict of interest. The reviewer, AK, declared a past co-authorship with one of the authors, CB, to the handling editor.
